# Increased iron content in the heart of the *Fmr1* knockout mouse

**DOI:** 10.1007/s10534-021-00320-1

**Published:** 2021-06-05

**Authors:** Karo Talvio, Katja M. Kanninen, Anthony R. White, Jari Koistinaho, Maija L. Castrén

**Affiliations:** 1 Faculty of Medicine, Physiology, University of Helsinki, P.O. Box 63, 00290 Helsinki, Finland; 2 A.I. Virtanen Institute for Molecular Sciences, University of Eastern Finland, Kuopio, Finland; 3 Department of Pathology, University of Melbourne, Melbourne, VIC Australia; 4 Cell and Molecular Biology, QIMR Berghofer Medical Research Institute, Brisbane, QLD Australia; 5 Neuroscience Center, HiLIFE, University of Helsinki, Helsinki, Finland

**Keywords:** Trace elements, Metals, Autism, Fragile X syndrome

## Abstract

Trace elements have important functions in several processes involved in cellular homeostasis and survival. Dysfunctional metal ion homeostasis can make an important impact on cellular defence mechanisms. We assessed the concentrations of 23 trace minerals in different tissues (brain, spleen, heart and liver) of *Fmr1* knockout (KO) mice that display the main phenotype of Fragile X syndrome (FXS), an intellectual disability syndrome and the best-known monogenic model of autism spectrum disorder (ASD). Altogether, seven minerals—Cu, Fe, K, Mg, Mn, Na, and P—were above the detection limit with the analysis revealing increased iron content in the heart of *Fmr1* KO mice. In addition, levels of iron were higher in the cerebellum of the transgenic mouse when compared to wild type controls. These results implicate a role for dysregulated iron homeostasis in FXS tissues and suggest that defective iron-related mechanisms contribute to increased tissue vulnerability in FXS.

**Supplementary Information:**

The online version contains supplementary material available at 10.1007/s10534-021-00320-1.

## Introduction

Fragile X syndrome (FXS) is an intellectual disability syndrome with a prevalence of around 1/4000 in males and 1/6000–8000 in females (Crawford et al. 1999). The FXS neurobehavioral phenotype includes abnormalities in speech development and communication, social behaviour, sensory reactivity, attention, arousal, and activity levels (Hagerman et al. 2010). The vast majority of FXS males display some behaviours characteristic of individuals with autism spectrum disorder (ASD) with some (~ 10–30%) fulfilling the standardised criteria for ASD (Brown et al. 1986). FXS is a monogenic disorder caused by the absence of FMR1 protein (FMRP) and the *Fmr1* knockout (KO) mouse recapitulates the human FXS phenotype. In most FXS patients, a CGG triple repeat expansion comprising > 200 repeats leads to transcriptional silencing of the *FMR1* gene (O’Donnell and Warren 2002). The length of the repeated CGG sequence of the *FMR1* gene in the range of 6–44 repeats is normal, whereas 45–54 repeats is intermediate or grey area, and 55–200 repeats is considered as premutation (Verkerk et al. 1991; Penagarikano et al. 2007). The premutation causes increased *FMR1* mRNA levels that associates with slightly reduced FMRP expression and does not result in any neurodevelopmental syndrome, but can lead to the onset of Fragile X-associated Tremor/Ataxia Syndrome (FXTAS) after the age of 50 years (Loesch and Hagerman 2012).

An inappropriate intake or absorption of trace elements is associated with a variety of health problems. Altered metal homeostasis is linked to a number of disorders and heavy metal exposure is presented as a potential risk factor for neurodevelopmental disorders (Froehlich et al. 2011; Scassellati et al. 2012; Kanninen et al. 2013). It has been suggested that metal deficiency and/or toxic metal burdens may epigenetically contribute to the manifestation of ASD (Yasuda et al. 2013). However, several studies searching changes of trace elements/metals in ASD have generated inconsistent results with high individual variation in levels of minerals (Saghazadeh et al. 2017; Tseng et al. 2018; Curtin et al. 2018; Austin et al. 2019; Hassan et al. 2019). Studies of genetic mouse models of neurodevelopmental diseases could provide important additional information about the relationship of trace metal balance and ASD.

Metal ions are tightly linked to the redox state of a cell. Oxidative stress is elevated in the brain of *Fmr1* KO mice (El Bekay et al. 2007), but tissue trace element homeostasis has not been previously examined in FXS. We investigated biometal concentrations in different tissues of the *Fmr1* KO mice. Using sensitive inductively coupled plasma mass spectrometry (ICP-MS) to analyse trace elements, seven minerals were reliably detected in mouse tissues and iron content proved to be increased in the heart of the *Fmr1* KO mouse.

## Methods

### Mice

*Fmr1* KO mice (B6.129P2-Fmr1tm1/Cgr/J, Jackson Laboratory, Bar Harbor, ME) and their wild type (WT) littermates were used at the age of 4–5 months. Genotyping by tail-PCR was performed as previously described (Bakker et al. 1994). Mice were housed in groups and subjected to a 12-h light–dark cycle with access to food and water ad libitum. All animal experiments were done in accordance with the guidelines of the National Institute of Health Guide for the Care and Use of Laboratory Animals and carried out using protocols approved by the Experimental Animal Ethical Committee of Southern Finland.

### Mouse tissues

For tissue collection, mice were anaesthetised with carbon dioxide and sacrificed by cervical dislocation. Brains, spleens, hearts, and livers were collected. Cerebellums and the ventral regions of the cerebral cortex were further dissected. The tissue pieces were weighed. There were no differences of average wet weights (p < 0.05) between control and FXS tissue samples (Table 1). All tissues were snap frozen and stored at − 80 °C until use.

**Table 1:** Sample preparation

Tissue	N	Average weight (mg) WT	Average weight (mg) *Fmr1* KO	HNO_3_ volume (µl)	Reduced volume (µl)	Dilution factor	Vol of digest (µl)
Cerebellum	12	63 ± 5.9	52 ± 8	100	150	21	50
Cortex	12	140 ± 5.2	140 ± 11	100	135	21	50
Heart	14	82 ± 8	59 ± 7	150	210	21	50
Liver	12	170 ± 20	160 ± 17	300	470	51	30
Spleen	13	56 ± 6.9	70 ± 4.4	150	210	21	50

Sample weights are mean ± SEM

### Mass-spectrometry analysis

The tissue concentrations of trace elements were measured by an established and fully validated method using ICP-MS as described previously (Maynard et al. 2002; Kanninen et al. 2013). Due to technical problems, four samples were not processed (*Fmr1* KO cortex, WT liver and both *Fmr1* KO and WT cerebellum). Briefly, weighed tissue samples were lyophilised, digested in 100–300 µl of 65% nitric acid (Merck, Kilsyth, Victoria, Australia) overnight at room temperature (RT), and heated for 20 min at 90 °C. Then an equivalent volume of 30% hydrogen peroxide (30% Aristar, BDH) was added and samples were incubated for 30 min at RT, followed by 15 min at 70 °C. The average reduced volume was determined, and the samples were diluted with 1% nitric acid diluent using dilution factors as shown in Table 1. Volume of digest was the amount of the digested sample that was used to make the dilution.

Measurements were made with an Agilent 7700 series ICP-MS instrument (Agilent Technologies, Santa Clara, CA, USA) under routine multi-element operating conditions using helium as cell gas. The instrument was calibrated using 0, 5, 10, 50, and 100 ppb of certified multi-element standard calibration solutions (ICP-MS-CAL2-1, ICP-MS-CAL-3, and ICP-MS-CAL-4; Accustandard, New Haven, CT, USA) for the range of the elements and 200 ppb of Yttrium (Y89) was used as internal control (ICP-MS-IS-MIX1-1, Accustandard). Three media blanks were used to determine detection limits. Conversion of readings in ppb was performed as follows: (μg/g) = (raw ppb value × dilution factor × reduced digest volume)/(tissue wet weight g). Samples were analysed in triplicate and median values were used for analyses. Results are expressed in micrograms of metal per gram of wet (µg/g wet wt). The concentration of 23 minerals and trace elements were assessed in mouse heart, liver, spleen, and brain tissues. Altogether, 7 trace elements (Cu, Fe, K, Mg, Mn, Na, and P) were above detection limits in our set of samples (Table 2), whereas 16 trace elements (Al, B, Ba, Ca, Cd, Co, Cr, Li, Mo, Ni, Rb, Ru, Se, Sr, Ti, and Zn) could not be reliably detected.

**Table 2:** The absolute metal content in the set of WT tissue samples of average size and concentrations of the medium and acid blanks

Metal	Cortex	Cerebellum	Liver	Spleen	Heart	Media blank (µg/g)	Acid blank (µg/g)
Na (µg)	210	96	130	38	98	25	32
Mg (µg)	14	7.5	36	9.2	14	0.69	0.31
P (µg)	330	190	530	180	170	4.2	2.5
K (µg)	330	160	500	180	160	4.1	5.4
Mn (ng)	29	21	120	8.2	31	0.013	0
Fe (ng)	910	540	12,000	26,000	3500	0.29	0
Cu (ng)	300	200	720	36	360	0.076	0.0016

### Statistical analysis

Statistical significance between KO and WT mouse tissues with each variable was determined with the Student’s *t*-test. The principal component analysis (PCA) was performed with all 35 metal variables (7 elements in 5 tissues). In the analysis, two WT mice were excluded due to several missing values and the missing values were replaced with the means when maximum of two tissues were missing. All statistical analyses, including linear correlations across all variables, were carried out using IBM SSPS Statistics. Data are shown as mean ± standard deviation. The criterion for significance was set to p < 0.05.

## Results

**Fig. 1:**
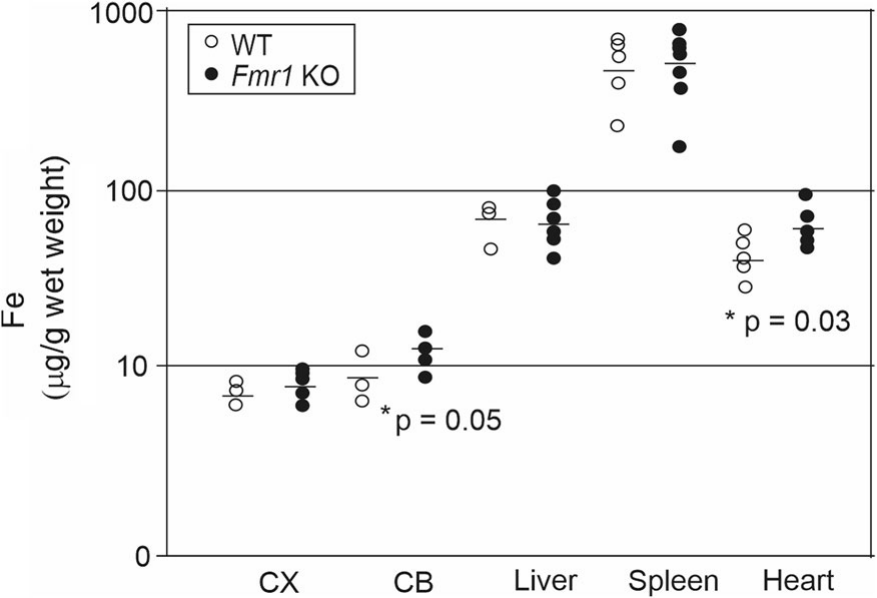
Concentration of iron across tissues. Values for individual *Fmr1* KO and WT mice are plotted. *p ≤ 0.05

Mean metal concentrations of WT and KO mouse tissues are presented in Table 3. Most significantly, iron was increased in the hearts (p = 0.033) of the KO mice (Fig. 1). The highest iron content was seen in spleen and the levels were relatively low in the brain tissues. An increase in iron content in the cerebellum of the *Fmr1* KO was found at the marginal level of significance (p = 0.052). The methodology suffered from low power and these statistical significances should be considered in the context of 35 individual comparisons (7 metals in 5 tissues) between KO and WT mice (Bonferroni correction for a typical α = 0.05 with 35 comparisons p < 0.0014, and the family-wise error rate for 35 comparisons at α = 0.05 was 0.83).

**Table 3:** Concentrations of different metals in heart, spleen, liver, cerebellum, and cortex of *Fmr1* KO mice compared to WT controls

Metal		Cortex	Cerebellum	Liver	Spleen	Heart
Na (µg/g)	WT	1500 ± 97	1500 ± 360	790 ± 110	680 ± 220	1200 ± 230
	*Fmr1* KO	1500 ± 230	1900 ± 330	730 ± 73	590 ± 160	1400 ± 380
	p	0.83	0.21	0.39	0.41	0.19
Mg (µg/g)	WT	100 ± 13	120 ± 36	210 ± 19	160 ± 51	170 ± 48
	*Fmr1* KO	95 ± 18	140 ± 38	210 ± 31	150 ± 34	210 ± 48
	p	0.61	0.4	0.8	0.51	0.16
P (µg/g)	WT	2300 ± 280	3100 ± 980	3100 ± 240	3200 ± 1000	2100 ± 630
	*Fmr1* KO	2300 ± 420	3600 ± 1000	3000 ± 430	2900 ± 720	2500 ± 550
	p	0.82	0.41	0.5	0.55	0.23
K (µg/g)	WT	2400 ± 300	2500 ± 810	2900 ± 190	3200 ± 1000	2000 ± 640
	*Fmr1* KO	2300 ± 420	3100 ± 800	3100 ± 420	2900 ± 670	2700 ± 600
	p	0.67	0.35	0.53	0.49	0.069
Mn (µg/g)	WT	0.21 ± 0.014	0.33 ± 0.093	0.71 ± 0.05	0.15 ± 0.064	0.38 ± 0.083
	*Fmr1* KO	0.19 ± 0.039	0.36 ± 0.097	0.79 ± 0.13	0.16 ± 0.05	0.49 ± 0.15
	p	0.39	0.69	0.14	0.69	0.11
Fe (µg/g)	WT	6.6 ± 0.91	8.6 ± 2.6	70 ± 15	460 ± 200	43 ± 11
	*Fmr1* KO	7.6 ± 1.5	13 ± 2.8	66 ± 19	510 ± 190	62 ± 17
	p	0.18	0.052	0.74	0.7	0.033 *
Cu (µg/g)	WT	2.2 ± 0.27	3.2 ± 1.7	4.2 ± 0.93	0.64 ± 0.21	4.4 ± 1.2
	*Fmr1* KO	2 ± 0.44	4.4 ± 1.1	4.2 ± 1.2	0.55 ± 0.13	5.5 ± 1.4
	p	0.39	0.31	0.96	0.44	0.16

*p < 0.05

**Fig. 2:**
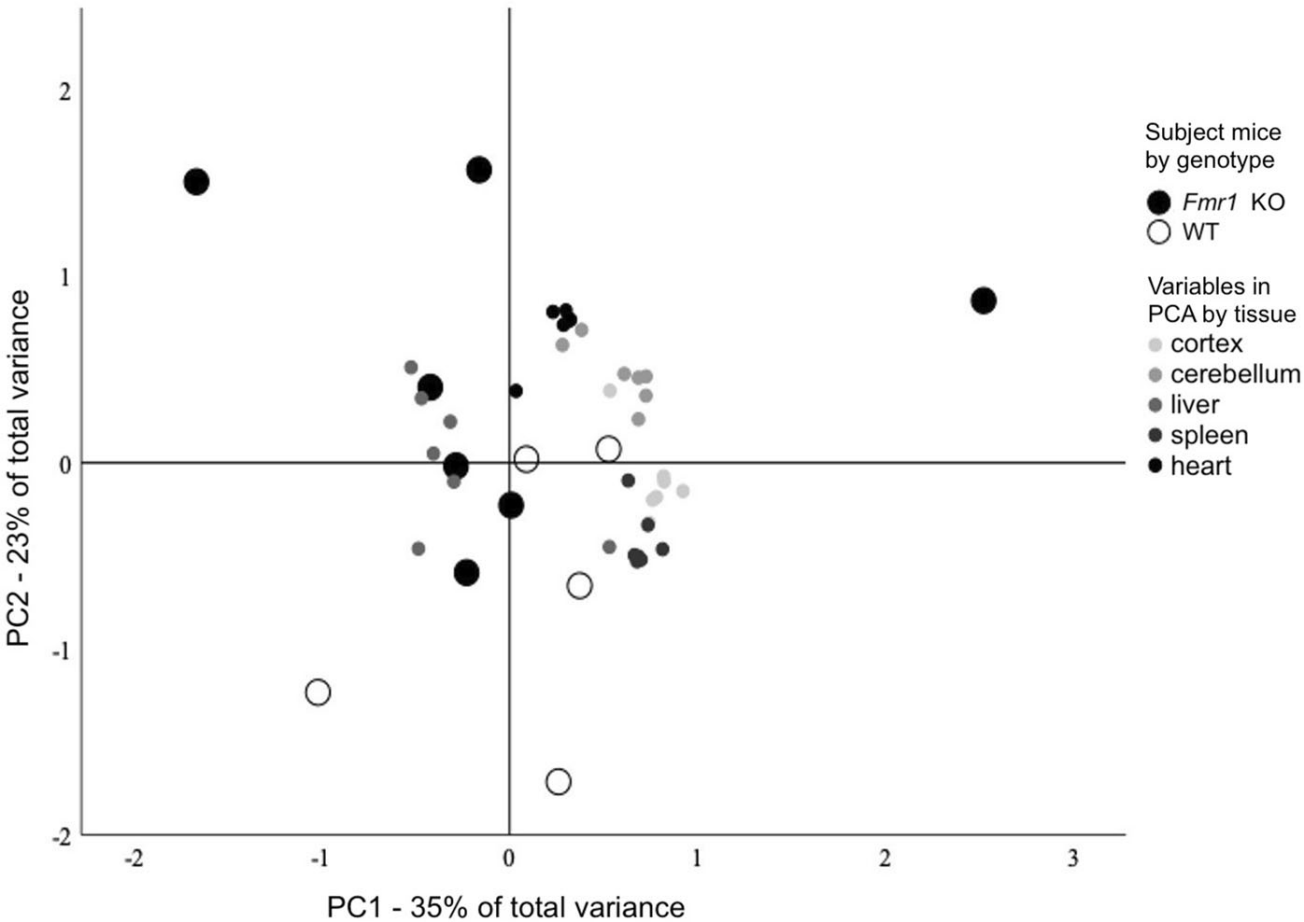
Principal component analysis (PCA) that was used to classify *Fmr1 *KO and WT mice based on metal content of seven different metals in five tissues is shown with respect to PC1 and PC2. KO and WT mice segregated across PC2 (p = 0.031) that explained 35% of the total variance and correlated with heart variables. Variables are coded by colour with respect to tissue studied

To evaluate the complete dataset for pattern of variations, we used the PCA. Three components were extracted, explaining a total of 73% of the total variance; PC1 explained 35%, PC2 23%, and PC3 15%. PC1 correlated closely with brain and spleen variables. PC2 correlated with heart variables and PC3 was influenced mostly by liver variables. T-tests with respect to the genotype on each extracted principal component showed that KO and WT mice segregated across PC2 (p = 0.031), but not across PC1 (p = 0.9) or PC3 (p = 0.99), implicating altered biometal homeostasis in the heart of the *Fmr1* KO mouse. Subject mice by genotype and variables by the tissue of the variable are shown in Fig. 2.

Brain and liver metal levels measured in the present study were comparable to previously reported measurements in mouse and rat tissues (SATO et al. 1996; Schneider et al. 2014; Garza-Lombó et al. 2018). In our analysis, within a tissue, metal concentrations generally correlated positively, but negatively with the mass of the tissue sample. Furthermore, we observed that Mg correlated strongly (r > 0.95) with P and K in most tissues, whereas Cu correlated strongly with P, Mg and K in spleens and hearts. A correlation matrix is provided in Online Resource 1.

## Discussion

The present study demonstrated elevations of iron levels in heart and cerebellum of the *Fmr1* KO mouse. Iron is a redox-active metal and an essential component of many proteins involved in biological defence mechanisms against oxidative stress (Crichton and Pierre 2001). A moderate increase in stress status has been previously reported in the adult *Fmr1* KO mouse brain, reflecting a deficient antioxidant system together with higher levels of reactive oxygen species, nicotinamide adenine dinucleotide phosphate (NADPH) -oxidase activation, lipid peroxidation and protein oxidation (El Bekay et al. 2007). Furthermore, studies of human *FMR1* premutation fibroblasts have revealed mitochondrial chain dysfunction and abnormally increased reactive oxygen species (ROS) production, which responded to chelation of iron with desferrioxamine mesylate (DFO) (Napoli et al. 2011). Since the *FMR1* premutation alters *FMR1* transcript expression and slightly reduces FMRP levels, the findings are consistent with involvement of FMRP in the regulation of iron balance.

FMRP has previously been implicated in protective mechanisms against injury in heart (Bao et al. 2018). Overexpression of FMRP was found to alleviate oxidative stress and apoptosis in damaged cardiomyocytes. Many FXS patients suffer from dilatation of aortic root and mitral valve prolapse that have been linked to connective tissue dysplasia. There is also evidence that sympathetic activity augments cardiac activity and output in FXS more than in healthy controls (Sreeram et al. 1989). A 1.4-fold increase in iron content in the heart tissue of *Fmr1* KO mouse provides evidence of pathological cellular processes, but its impact on heart function remains to be investigated. The extent of iron toxicity depends on localisation of the iron complex within the cell e.g. cytosolic vs. lysosomal, its biochemical form, and the cellular content of a wide range of antioxidants and cytoprotective enzymes that can prevent the generation and propagation of free radical species. Mitochondrial iron accumulation in cardiac tissue and brain is associated with Friedrich ataxia (FRDA). The excess iron exists as highly localized multifocal aggregates rather than a diffuse pattern in the tissue of FRDA patients and iron-mediated toxicity is not well understood (Llorens et al. 2019).

Region-specific accumulation of iron can particularly contribute to the pathophysiology of brain diseases due to the decreased ability of neuronal cells to respond to oxidative stress. Increased iron levels have been linked to abnormalities of cerebellar myelination (Beltrán-Navarro et al. 2012; Klocke et al. 2018; Fernández et al. 2019). Accumulation of iron in the cerebellum of the *Fmr1* KO mice is especially interesting considering the cerebellar changes and altered firing rate of neurons in cerebellar circuitry of *Fmr1* KO mice (Koekkoek et al. 2005). Cerebellar vermis is essential in gating of sensorimotor reactions (Leaton and Supple 1986), and impaired acoustic startle reflex in human and mouse models of FXS also supports involvement of cerebellar defects in FXS. Cerebellum was not studied as a separate brain region in the oxidative stress study of *Fmr1* KO mice by el Bekay et al. (2007). Mild cerebellar accumulation of iron was previously shown in a subset of *FMR1* premutation carriers with FXTAS (Rogers et al. 2016), who display defective iron and zinc metabolism (Napoli et al. 2011).

Biometal supplements are used without medical prescription and are often tested as treatment of neurodevelopmental disorders when no other treatment is available. Current research data do not provide sufficient information to formulate recommendations for use of trace elements/metals (Lyall et al. 2014; Devilbiss et al. 2017). Iron is important for normal behavioural development and its deficiency is often seen in ASD patients (Latif et al. 2002). However, altered iron levels in ASD may be primarily of environmental origin and not directly linked to ASD (Reynolds et al. 2012). Mouse models offer possibilities to investigate disease-related biometal homeostasis and to evaluate the effectiveness of treatments. It is important to note that accumulation of trace elements may increase the risk of tissue damage. Our observation that heart iron content was increased in the FXS mouse may have clinical impact, because increased cardiac iron levels can lead to iron overload cardiomyopathy, a potentially lethal condition. Furthermore, our study suggests that brain region-specific alterations of iron exist in FXS. Altogether, the present findings showing accumulation of iron in distinct *Fmr1* KO mouse tissues are fundamental and promote further research to evaluate role of biometals in FXS and other neurodevelopmental disorders.

## Supplementary Information

Below is the link to the electronic supplementary material.

Supplementary file1 (XLSM 29 kb)
